# First Specific Detection of Mammalian Orthoreovirus from Goats Using TaqMan Real-Time RT-PCR Technology

**DOI:** 10.3390/vetsci11040141

**Published:** 2024-03-22

**Authors:** Li Mao, Xia Li, Xuhang Cai, Wenliang Li, Jizong Li, Shanshan Yang, Junjun Zhai, Sizhu Suolang, Bin Li

**Affiliations:** 1 Institute of Veterinary Medicine, Jiangsu Academy of Agricultural Sciences, Nanjing 210014, China; 20100014@jaas.ac.cn (L.M.); 18780388526@163.com (X.L.); caixuhang1999@163.com (X.C.); kfliwenliang@163.com (W.L.); 20150044@jaas.ac.cn (J.L.); yangshanshan@caas.cn (S.Y.); 2 Key Laboratory of Veterinary Biological Engineering and Technology, Ministry of Agriculture, Nanjing 210014, China; 3 National Center for Engineering Research of Veterinary Bio-Products, Nanjing 210014, China; 4 Jiangsu Key Laboratory for Food Quality and Safety-State Key Laboratory Cultivation Base of Ministry of Science and Technology, Nanjing 210014, China; 5 College of Animal Science, Tibet Agricultural and Animal Husbandry University, Linzhi 860000, China; xzslsz@163.com; 6 Jiangsu Co-Innovation Center for Prevention and Control of Important Animal Infectious Diseases and Zoonoses, Yangzhou University, Yangzhou 225000, China; 7 College of Veterinary Medicine, Northwest A&F University, Xianyang 712100, China; 8 Shaanxi Province Engineering and Technology Research Center of Cashmere Goat, Yulin University, Yulin 719000, China; zhaijunjun1980@163.com

**Keywords:** mammalian orthoreovirus, TaqMan qRT-PCR, detection, goat, sheep

## Abstract

**Simple Summary:**

Mammalian orthoreovirus (MRV) infects many hosts, including humans and animals, in which it causes gastroenteritis and respiratory disease. In China, it has been found in domestic animals such as pigs, cattle, deer, and minks; however, MRV prevalence in small ruminants is still unknown, and a rapid, specific, quantification assay for clinical detection of animal samples is urgently needed. In this study, we established a TaqMan qRT-PCR method for MRV detection targeting the conserved L1 gene. The method was optimized, and the specificity, sensitivity, and repeatability were used to assess quality. We used this method for MRV detection in goat and sheep samples. The overall MRV prevalence was 8.2% (35/429), and the test showed a significantly higher sensitivity than RT-PCR and virus isolation. MRV clearly showed seasonal prevalence, with a high positive rate of infections in goats and sheep from September to April. Small ruminants under two months of age were susceptible to MRV infection. This study investigated the epidemiological characteristics of MRV infection and provides the first evidence of MRV infection in sheep and goats in China, broadening our knowledge of MRV hosts in this country.

**Abstract:**

Mammalian orthoreovirus (MRV) infections are ubiquitous in multiple mammalian species including humans, and mainly causes gastroenteritis and respiratory disease. In this study, we developed a rapid and sensitive TaqMan qRT-PCR method for MRV detection based on the primers and probe designed within the conserved L1 gene. The qRT-PCR assay was evaluated for its sensitivity, specificity, efficiency and reproducibility. It was found that the detection sensitivity was equivalent to 10 DNA copies/μL, and the standard curves had a linear correlation of R^2^ = 0.998 with an amplification efficiency of 99.6%. The inter- and intra-assay coefficients of variation (CV%) were in the range of 0.29% to 2.16% and 1.60% to 3.60%, respectively. The primer sets specifically amplified their respective MRV segments and had the highest detection sensitivities of 10^0.25^ TCID_50_/mL with amplification efficiencies of 99.5% (R^2^ = 0.999). qRT-PCR was used for MRV detection from samples of sheep, goats, and calves from four regions in China, and the overall MRV prevalence was 8.2% (35/429), whereas 17/429 (4.0%) were detected by RT-PCR and 14/429 (3.3%) by virus isolation. The qRT-PCR assay showed significantly higher sensitivity than RT-PCR and virus isolation. Results from an epidemiological survey indicated that the positive rate of MRV in rectal swabs from sheep and goats tested in Shaanxi, Jiangsu, and Xinjiang were 9/80 (11.3%), 12/93 (12.9%) and 14/128 (10.9%), respectively. In goats and sheep, MRV prevalence was obviously associated with season and age, with a high positive rate of more than 8% during September to April and approximately 13% in small ruminant animals under two months of age. This is the first instance of MRV infection in sheep and goats in China, thus broadening our knowledge of MRV hosts. Consequently, primer optimization for qRT-PCR should not only prioritize amplification efficiency and specificity, but also sensitivity. This assay will contribute to more accurate and rapid MRV monitoring by epidemiological investigation, viral load, and vaccination efficacy.

## 1. Introduction

Mammalian orthoreovirus (MRV) is a non-enveloped, segmented, and double-stranded RNA virus belonging to the genus *Orthoreorivus* in the *Reoviridae* family [1]. Its genome length is approximately 23.5 kb and contains 10 genetic segments of three size classes: three large (L1, L2 and L3), three medium (M1, M2 and M3), and four small (S1, S2, S3 and S4) segments. According to the neutralization assays, four major serotypes of MRV have been identified: Type 1 (MRV1), Lang (T1L); Type 2 (MRV2), Jones (T2J); Type 3 (MRV3), Dearing (T3D); and a putative Type 4 (MRV4), Ndelle (T4N). MRV is widespread and infects a broad range of mammals including humans [2], pigs [3], red deer [4], bats [5], cattle [6], minks [7], dogs [8], wild boar [9], chamois [10], and civets [11]. 

In humans and animals, MRV has been identified as a cause of mild gastroenteritis and respiratory disease, ranging from symptomatic to asymptomatic. In Malaysia, MRV has been linked to respiratory infections in patients [12,13]. In pigs, MRV3 has been associated with fever, diarrhea, and respiratory disease in Europe, North America, and Asia [3,14,15,16]. Additionally, MRV2 and MRV3 have been found to cause necrotizing encephalopathy and meningitis in the United States and Europe [2,17]. Furthermore, MRV1 and MRV3 have been shown to induce disease in the central nervous system in mice [18,19,20], indicating that MRV may be responsible for more serious illnesses.

Reassortment is usually observed in animals infected with various MRV lineage strains and has been confirmed in hosts including voles, partridges, bats, and calves. Subsequent research, as determined by sequence and evolutionary analysis, has revealed that the reassorted segments might originate from various hosts [21,22,23]. The segmented structure of MRVs allows for reassortment when one host is infected with multiple different strains, leading to the emergence of new strains and evolutionary advancement. This suggests that MRVs may be capable of cross-species transmission. However, reassortment has brought challenges to the establishment of pathogenic nucleic acid detection methods. 

Currently, there are several reverse transcriptase polymerase chain reaction (RT-PCR) techniques available for the detection of MRVs, which can be combined with sequencing technology to verify the presence of the virus genome. The detection of MRVs in bat guano in Slovenia was detected by broad-range RT-PCR targeting the conserved region in the L3 gene, and 44 out of 443 samples (9.9%) were positive for MRV [24]. Several studies developed MRV RT-PCR methods targeting the L1 gene, which expresses the viral RNA-dependent RNA polymerase and is conserved among different reovirus strains. A nested PCR assay specific to reoviruses was established by Leary et al. and used to detect MRV in ungulates in Northern Italy. MRV was detected in a total of 110 samples, and 56 nasal and 31 ocular swabs from dogs with diarrhea or nasal discharge tested positive [25]. Another consensus nested PCR method was conducted and used to detect reoviruses of three different virus species, including reptilian, avian, and mammalian orthoreoviruses [26,27]. To identify MRV 3 in Italian bats [28] and pigs in Zambia [8,26], RNA samples were tested using specific primers based on the conserved L1 gene, and MRV genomes were detected in 19.7% (29/147) of fecal samples collected from pigs. An RT-PCR protocol was also designed to detect MRV in fecal samples from neonatal alpacas and llamas, and MRV was the second most prevalent virus, being detected in 50% of samples [29]. However, rapid and specific diagnosis of MRV and quantification of viral load is urgently needed for clinical detection of animal samples. This study aimed to establish a one-step real-time qRT-PCR method for the precise detection and quantification of MRV, which can be used as a beneficial tool for diagnosis, epidemiological surveillance, and future vaccination efficacy evaluation.

## 2. Materials and Methods

### 2.1. Design of Primers and TaqMan Probe

Various locations and hosts of six MRV L1 genes and highly conserved sequences in MRV genome were downloaded from GenBank (OQ789607, MN639757, MK092964, LC613209, LC752173, MW718862, Table S1), and aligned by ClustalW in MEGA 6.06. PCR primers and a TaqMan probe (Table S2) were designed with Primer Premier version 5.0 (Premier Biosoft, Palo Alto, CA, USA, 1999) based on the results of nucleotide comparison to specifically detect MRV, targeting the conserved L1 sequences among the different strains (Figure S1). The probes were labelled at their 5′ and 3′ ends with FAM and Black Hole Quencher-1 (BHQ-1), respectively.

### 2.2. Construction of L1 Standard Plasmid

The standard curve, specificity, and sensitivity of the MRV-specific assay was assessed by a recombinant plasmid DNA with the L1 segment. The plasmid standards were prepared as follows: a fragment of 97 bp was obtained from MRV JS6 isolate (stored in our laboratory) using the designed primer pairs qMRV-F and qMRV-R (Table S2); the target band was recycled, purified, cloned into the pMD18-T vector (TaKaRa, Dalian, China), and then transformed into *Escherichia coli* DH5α competent cells (Transgen, Beijing, China). The separated colonies were identified by PCR with qMRV-F and qMRV-R primers and sequenced by Beijing Tsingke Biotech Co., Ltd. (Beijing, China). The MRV-positive plasmids (pMRV) extracted from *Escherichia coli* DH5α were cultured overnight. Subsequently, purified pMRV plasmids were quantified with a NanoDrop 2000 spectrophotometer, and the copy numbers were calculated by the following formula: Y (copies/μL) = 6.02 × 10^23^ × X (ng/μL) × 19^−9^/(length of RNA [bp] × 340). Serial 10-fold dilutions of plasmids were made in nuclease-free water to obtain 10^8^–10^0^ copies/μL of pMRV genome for qPCR. The prepared pMRV was stored at −20 °C until use.

### 2.3. Virus Isolation

MRV-positive nasal and rectal swab samples were prepared for three freeze–thaw cycles and then centrifuged at 12,000 rpm for 20 min. The supernatants were filtered through 0.22 μm filters to avoid bacterial contamination and then inoculated onto a monolayer of Vero cells in 24-well culture plates. The cells were observed for 3–5 days and blind passaged three more times. The cell cultures were harvested until 90% of cytopathic effects (CPE) were present, and then the cultures were frozen at −70 °C for later use.

### 2.4. RNA Extraction and RT-PCR Amplification

Total RNA was extracted from supernatant of cell cultures or swabs samples using RNA extraction kit (Vazyme Biotech Co., Ltd., Nanjing, China) according to the manufacturer’s instruction. A 20 μL RT-PCR mix was prepared with Easyscript one-step RT-PCR supermix (Transgen, Bio, Inc., Beijing, China) and contained 10 μL of 2 × R-Mix buffer, 0.5 μM of each primer (MRV-L1F and MRV-L1R, Table 1), 0.5 μL of E-Mix, and 4 μL RNA. The RT-PCR was performed with the following protocol: reverse transcription at 45 °C for 40 min, initial denaturation at 95 °C for 5 min; followed by 35 cycles at 95 °C for 30 s, 50 °C for 30 s, and 72 °C for 45 s; and a following final extension at 72 °C for 7 min. Amplification products of 560 bp were detected by 1.2% agarose gel.

### 2.5. Optimization of TaqMan qRT-PCR

The TaqMan qRT-PCR was performed with an ABI Step One system (Applied Biosystems, Foster City, CA, USA) for all assays using the HiScript II One Step qRT-PCR Probe Kit (Vazyme Biotech Co., Ltd., Nanjing, China) on a final volume of 20 μL, which included 100 nM of each primer and probe, 4 μL of RNA template and deionized distilled water. The TaqMan qRT-PCR conditions were as follows: reverse transcription at 50 °C for 5 min, initial denaturation at 95 °C for 30 s, 40 cycles at 95 °C for 10 s, and final extension at 60 °C for 34 s. PCR-grade water as negative control and the plasmid (pMRV) as positive control were employed in the TaqMan RT-qPCR experiments.

### 2.6. Method Specificity, Sensitivity and Repeatability

To assess the limit of detection (LoD), 10-fold dilutions of the quantified pMRV, containing 10^8^ to 10^0^ DNA copies/μL, were examined by RT-PCR and TaqMan RT-qPCR as described above. To evaluate the reproducibility and repeatability of the real-time RT-PCR, 10^8^ down to 10^0^ pMRV dilutions were tested in triplicate in three independent experiments. To validate the robustness of the assay, repeating experiments were performed with three real-time instruments (ABI 7500, ABI Step One thermo-cycler, ABI QuantStudio6, Thermofisher, Emeryville, CA, USA) by testing the 10-fold dilutions of pMRV. The LoD was also evaluated by testing serially diluted MRV virions (1 × 10^0.25^ TCID_50_/mL to 1 × 10^6^ TCID_50_/mL). Subsequently, using plasmid and positive samples as a template, the reproducibility of the TaqMan qRT-PCR was evaluated with three repeats. The mean and standard deviations (SD) and coefficients of variation (CVs) for quantification cycle (Ct) values of intra- and inter- assay were calculated in three independent runs. 

The assay specificity was tested by analyzing RNA or DNA samples extracted from previously known representative viruses of goats, including five MRV strains (MRV strains F2B, SY12, JS6, JS1 and XJ23), and *peste des petits ruminants* virus (PPRV), adenovirus (AdV), bovine viral diarrhea virus (BVDV), caprine enterovirus (CEV), caprine influenza virus type 3 (CPIV3), bovine rotavirus (BRV), and coronavirus (CoV) stored in our lab. The experiments were analyzed to make sure there were no false positive results or cross-reactions within pathogens from the same host species. Positive and negative controls were present in each assay.

### 2.7. MRV Detection in Clinical Samples

To investigate MRV prevalence in ruminant animals, 391 rectal swabs and 38 nasal swabs were collected from goats, sheep, and calves from four regions, Shaanxi, Jiangsu, Anhui, and Xinjiang, and detected by qRT-PCR, RT-PCR, and virus isolation, as established above. Prior to sample collection, the owners of the animals were contacted, and their permission was obtained. The experiments were performed strictly according to the guidelines of Jiangsu Province Animal Regulations (Government Decree No. 45). Informed consent regarding the testing of samples with the above methods was provided for all owners, and the owners agreed to the use and disclosure of questionnaire data. The collection time and location of samples, and the age and symptoms of animal, were recorded in detail. Rectal and nasal swab samples from all enrolled animals were collected by trained staff at admission and following standard operating procedures, and immediately transferred to the lab for virus detection.

### 2.8. Statistical Analysis

Standard curves for serially diluted varied dilutions were generated in Microsoft 365 Excel™ (Microsoft, Redmond, WA, USA, https://www.microsoft.com/en-us/microsoft-365/excel, accessed on 28 January 2024). The linear regression equation (R^2^) for each standard curve was used to determine the primer amplification efficiency (E). Microsoft Excel™ was used to determine the mean, SD, and percentage (%) of CV for intra- and inter-assay variability analysis as a measure of reproducibility.

## 3. Results

### 3.1. Standard Curve of qRT-PCR

**Figure 1:**
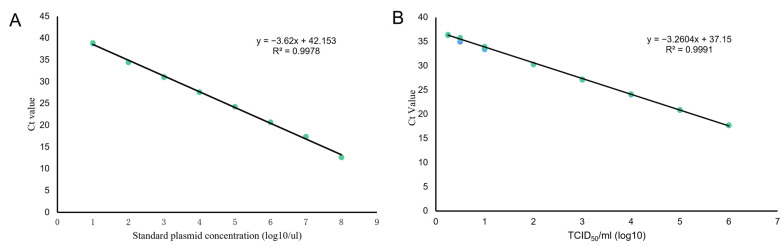
Linearity and analytical sensitivity of the one-step TaqMan RT-PCR for MRV. (**A**) Standard curve obtained with 10-fold serial dilutions (1 × 10^8^ to 1 × 10^1^) of MRV standard plasmid, and the equation of the curve was as follows: y = −3.62x + 42.153; R^2^ = 0.998; amplification efficiency E = 99.6%. (**B**) Standard curve constructed with serial dilutions (1 × 10^6^ to 1 × 10^0.25^ TCID50/mL) of MRV standard RNA, and the equation of the curve was as follows: y = −3.26x + 37.15; R^2^ = 0.999; amplification efficiency E = 99.5%. Each dilution was performed in triplicate.

The 10-fold diluted pMRV standard plasmid was used to evaluate the linearity, efficiency and dynamic range of the TaqMan qRT-PCR assay. Quantitative analysis identified the LoD of 10^3^ copies of pMRV for RT-PCR and 10 copies for TaqMan qRT-PCR (Table S3, Figure 1A). The assay was shown to generate standard curve over a range of 8log units of plasmid copies/μL with a good linear correlation (R^2^ = 0.998), from 1 × 10^8^ to 1 × 10^1^, with an efficiency of 99.6%. The dynamic experiment was repeated on three real-time instruments (ABI QuantStudio6, ABI 7500, ABI Step One thermo-cycler, Thermofisher, CA, USA) to validate the robustness of the assay and determine the LoD values. Ct values obtained from the three independent trials were analyzed, the linear ranges extended from 8.0 to 1.0 log10 copies/μL with a good correlation (R^2^ > 0.998), and the Ct values remained linear to 10 copies/μL; the results were concordant between the three technical replicates (Table S3). The LoD was also evaluated by testing diluted MRV virions (from 1 × 10^0.25^ TCID_50_/mL to 1 × 10^6^ TCID_50_/mL) to construct the standard curve. The efficiency was estimated as E = 99.5% (R^2^ = 0.999), with a detected minimum virus concentration (sensitivity) of 1 × 10^0.25^ TCID_50_/mL (Table S4, Figure 1B). 

### 3.2. Repeatability of qRT-PCR

To evaluate the repeatability of the method, six pMRV templates were evaluated by detecting each in triplicate through inter- and intra-assay comparisons. The inter-assay SD and CV ranged from 0.09 to 0.51 and 0.29% to 2.16% respectively, whereas the intra-assay SD and CV ranged from 0.28 to 0.95 and 1.60% to 3.60% respectively (Table S5). These findings indicated high reproducibility of the assay. 

### 3.3. Specificity of TaqMan RT-qPCR

Using the different purified viral RNA and DNA as templates, the specificity of the TaqMan RT-qPCR assay was determined. The results showed that the TaqMan method only yielded positive results when MRV RNA was present. Additionally, no cross-reaction was observed with AdV, BVDV, CEV, CPIV3, PPRV, BRV, and CoV DNA/RNA (Table S6), which confirmed that the assay was specific. Similar results were obtained from three independent experiments.

### 3.4. MRV Detection in Clinical Samples

A total of 391 rectal swabs and 38 nasal swabs from goats, sheep, and calves from four regions were detected by virus isolation, RT-PCR, and qRT-PCR. The TaqMan qRT-PCR showed an overall prevalence of 8.2% (35/429), whereas 17/429 (4.0%) were positive by RT-PCR and 14/429 (3.3%) by virus isolation (Table 1). Positive MRV infections were found in sheep and goats in the Shaanxi, Jiangsu, and Xinjiang regions, with positive rates of 9/80 (11.3%), 12/93 (12.9%), and 14/128 (10.9%), respectively. The qRT-PCR assay showed greater sensitivity for MRV detection than RT-PCR and virus isolation did. 

**Figure 2:**
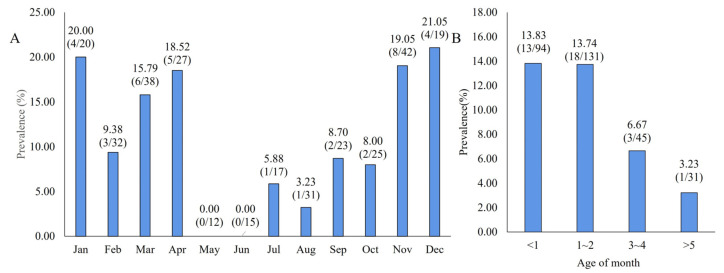
Epidemiological prevalence of MRV in goats and sheep. (**A**). Year-round seasonal MRV activity. (**B**). MRV prevalence detected in animals according to age group.

In addition, MRV was detected in goat and sheep populations year-round; a higher prevalence was found from September to the following April, and the positive rate of each month was more than 8%. Interestingly, the prevalence was significantly increased from November to the following April, which was during the winter and spring, and the positive rates were almost all above 15% (Figure 2A). It is speculated that MRV has seasonal prevalence. 

Furthermore, sampled animals were divided into four groups: under 1 month of age, 1–2 months, 3–4 months, and over 5 months of age. Animals under 2 months of age were more susceptible, and the positive rates of 1- and 2-month-olds were 13.83% and 13.74%, respectively, whereas the animals over 5 months of age were not susceptible (Figure 2B). This is the first report of MRV infecting sheep and goats in China, and there was little difference present in the positive rate in these three regions. However, rectal and nasal swabs from calves were detected as negative.

## 4. Discussion

The spread of infectious diseases caused by pathogens is a major challenge to the global public health system. MRVs infect a broad range of mammalian species, including humans, livestock, companion animals, and wildlife, and have been linked to neurological diseases, pneumonia, respiratory illness, and enteritis [20,30]. Additionally, MRVs are highly resilient and can remain in environmental sources such as surface water, seawater, and wastewater for an extended period of time [31,32]. MRV zoonotic transmission has been documented to occur through both direct and indirect contact [5]. To date, MRV infection has been discovered in many domestic animals in China, such as pigs, yaks, cattle [33,34], minks [7,35], and bats [36,37]. Therefore, it is imperative to be aware of MRVs as a novel infectious agent in both farm and wild animals in order to reduce the risk of cross-transmission to human beings and farm animals. In 2022, the number of goats and sheep in China reached about 320 million. With the situation that a variety of domestic animals have been infected with MRV, it is not known whether goats and sheep have been infected with MRV, and whether cross-infection occurred in small ruminants with other domestic animals. Because the viral genome is segmented, genetic reassortment and intragenic rearrangements are frequently present in MRVs across a wide host range, which could result in unpredictable biological characteristics and broaden the host species [10,38,39]. This genetic reassortment and intragenic rearrangement event can lead to the potential risk of species barrier breakdown, allowing MRVs to spread across species and infect humans as well as a broad range of other mammalian species, potentially resulting in a sudden surge of cases. Therefore, it is essential to detect MRVs and develop effective detection techniques.

To date, limited information exists regarding the epidemiology of MRV, and detection is traditionally carried out through RT-PCR. Several studies have been conducted to develop MRV RT-PCR methods, many of which have focused on the L1 gene, which expresses viral RNA-dependent RNA polymerase and is conserved among different reovirus strains. Despite the recent development of a series of RT-PCR methods, there are currently no qRT-PCR assays available for virus pathogen identification. The outbreak of MRV in animals and humans underscores the need to establish and optimize rapid and ideal diagnostic tools for early detection of potentially emerging viruses. The TaqMan qRT-PCR technique, which is based on viral RNA, is a reliable tool for the rapid detection of the causative agent of MRV infections. 

In this study, a highly sensitive and specific one-step qRT-PCR assay was developed for MRV detection based on the L1 gene and revealed no cross-amplification between viruses of different species. Good linearity was observed for a wide range (1 × 10^8^ to 1 × 10^1^ copies/μL) of a standard plasmid (E = 99.6%, R^2^ = 0.998). Additionally, the method was proven to be effective for detection of viral RNA extracted from cell culture supernatants with a range of 10^0.25^ TCID50/mL to 10^6^ TCID_50_/mL (E = 99.5%, R^2^= 0.999). Our assay yielded high precision between experiments and a wide dynamic range, as evidenced by the R^2^ values of 0.998 and 0.999, which indicate efficiencies of 99.5% to 99.6%, respectively. The samples tested in triplicate in different runs showed very low concentrations of MRV standard plasmid (10 copies/μL) and MRV titers (1 × 10^0.25^ TCID_50_/mL), which confirmed the assay’s sensitivity for MRV detection, even in animals with low-level viremia. The inter- and intra-assay CVs were found to be 0.29–2.16% and 1.60–3.60% (Table S3), respectively, which were all below the acceptable limit of 5%. Therefore, it can be concluded that the qRT-PCR method developed for MRV detection is highly reproducible and stable. In addition, the sensitivity experiment was repeated on three different real-time instruments, the CV values were analyzed <5%, which validated the robustness of the assay under different experimental conditions.

Real-time PCR has been widely used recently for clinical detection. Compared with conventional PCR, real-time PCR detects the samples in real time, with strong specificity and better sensitivity, and presents a visual representation of the amount of amplified products. Therefore, this technique was employed to assess its feasibility for use in MRV detection in clinical samples. A total of 429 rectal and nasal samples were subjected to TaqMan qRT-PCR, RT-PCR and virus isolation, which yielded an overall prevalences of 8.2% (35/429), 4.0% (17/429), and 3.3% (14/429), respectively (Table 1). The results revealed that qRT-PCR had a higher detection rate and displayed significantly higher sensitivity than RT-PCR and virus isolation. Importantly, MRV was detected and isolated from sheep in Shaanxi and Xinjiang and goats in Jiangsu with positive rates of 11.3%, 12.9% and 10.9%, respectively. This indicates that goats and sheep can be infected with MRV, and the infection rate is approximately 11%. 

Furthermore, the seasonal and age distributions of MRV infections in small ruminants were analyzed. MRV prevalence had obvious seasonality and was higher than 15% from November to April; there was obvious seasonal prevalence when the temperature was low and the winter and spring seasons changed. In addition, we also found that MRV prevalence was higher in animals under 2 months of age (about 13%), which indicated that the virus mainly infected young animals. 

This study demonstrated that MRV is endemic to China, and the known MRV host range was broadened to include goats and sheep, which was likely due to cross-transmission from other animal infections. These findings indicate that there is a lack of species barriers between domestic and wild animals. In addition, our research revealed that this virus was mainly present in rectal swabs; this suggests that infected animals are likely to shed the virus through feces, making it easier to detect the virus in rectal swabs. This emphasizes the significance of fecal treatment and disinfection in MRV prevention and control.

## 5. Conclusions

In conclusion, this study confirmed the successful establishment of a TaqMan qRT-PCR-based assay for MRV detection that is both fast and reliable. By this detection assay, the known host range of MRV was broadened to include goats and sheep and showed a prevalence of 8.2%. Additionally, we found that MRV showed obvious seasonal prevalence in winter and spring, and animals younger than 2 months of age were susceptible. Linking this method to sequencing and phylogenetic studies can be beneficial in epidemiological studies, allowing us to gain a better understanding of the prevalence, distribution and movements of certain MRV strains.

## Figures and Tables

**Table 1:** MRV epidemiological investigation using TaqMan qRT-PCR, RT-PCR, and virus isolation methods.

Samples	Location	Host	RT-PCR (Positive Rate)	qRT-PCR (Positive Rate)	Ct Range	Virus Isolation
Rectal swabs (*n* = 80)	Shaanxi	sheep	2/80 (2.5%)	9/80 (11.3%)	29.58–31.72	0/9
Rectal swabs (*n* = 93)	Jiangsu	goat	12/93 (12.9%)	12/93 (12.9%)	18.44–30.61	12/93 (12.9%)
Nasal swabs (*n* = 26)	Jiangsu	calf	0/26 (0)	0/26 (0)	/	0/26 (0)
Nasal swabs (*n* = 12)	Anhui	calf	0/12 (0)	0/12 (0)	/	0/12 (0)
Rectal swabs (*n* = 128)	Xinjiang	sheep	3/128 (2.3%)	14/128 (10.9%)	15.48–26.49	2/128 (14.3%)
Rectal swabs (*n* = 90)	Xinjiang	calf	0/90	0/90 (0)	/	0/90 (0)
Total			17/429 (4.0%)	35/429 (8.2%)		14/429 (3.3%)

## Data Availability

Data are contained within the article.

## Supplementary Materials

The following supporting information can be downloaded at: https://www.mdpi.com/article/10.3390/vetsci11040141/s1, Table S1. Deposited sequences used for primers design during this study. Table S2. Nucleotide sequences of primers and probe in L1 gene of MRV. Table S3. The Ct values obtained with 10-fold serial dilutions (1 × 10^8^ to 1 × 10^1^) of MRV standard plasmid by TaqMan RT-PCR method. Table S4. The Ct values obtained from serial dilutions (1 × 10^6^ to 1 × 10^0.25^ TCID50/mL) of MRV standard RNA by TaqMan RT-PCR method. Table S5. Intra-and inter-assay variability of the TaqMan RT-qPCR for MRV detection. Table S6. The Ct values of different viruses detected by TaqMan RT-PCR method. Figure S1. Sequence alignment of a conserved fragment of the L1 gene. PCR primers are marked in blue flame, and probe in red.
