# Supplementary material for: First Specific Detection of Mammalian Orthoreovirus from Goats Using TaqMan Real-Time RT-PCR Technology
Source: Vet Sci. 2024 Mar 22;11(4):141. doi: 10.3390/vetsci11040141 (PMC11054425; doi:10.3390/vetsci11040141)

Table S1. Used sequences for primers design during this study.

Accession number	Strain	Country	Isolated from	Isolation Date
OQ789607	SNU1	Korea	Bat	2021
MN639757.1	19/242	Germany	Bank vole	2019
MK092964.1	OV204	USA	Odocoileus virginianus	2016
LC613209	THK0325	Japan	wastewater	2020
LC752173.1	Kj22-33	Japan	Vespertilio sinensis	2022
MW718862.1	17-EF40	USA	Eptesicus fuscus	2017

Table S2. Nucleotide sequences of primers and probe in L1 gene of MRV.

Primers and probe	Nucleotide sequences (5'-3')
RT-PCR	
MRV-L1F	TTCACTCAGGCATTATCCGA
MRV-L1R	TCCGCTTCTGACTCCTGA
qRT-PCR	
qL1F	GTCTCAGGCTCGACAGATTAAG
qL1R	TGCAGAACGGGATCATATAAGG
probe	FAM-AGTTTGTGTTGGCGTTATTGGTGGC-TAMRA

Table S3 The Ct values obtained with 10-fold serial dilutions (1×10^8 to 1×10^1) of MRV standard plasmid by TaqMan RT-PCR method

	QuantStudio6			7500			Step One					
Log10/ul	Ct1	Ct2	Ct3	Ct1	Ct2	Ct3	Ct1	Ct2	Ct3	Mean	SD	CV%
1	38.867	38.812	38.924	38.904	38.96	38.901	39.578	39.272	39.365	39.054	0.281	0.72
2	34.466	34.51	34.445	34.575	34.522	34.516	34.907	34.989	35.095	34.669	0.253	0.73
3	31.055	31.048	31.051	31.534	31.544	31.628	31.553	31.659	31.476	31.394	0.263	0.84
4	27.577	27.583	27.615	27.613	27.589	27.592	28.087	28.288	28.273	27.802	0.316	1.14
5	24.231	24.254	24.238	24.245	24.257	24.233	24.635	24.763	24.746	24.400	0.239	0.98
6	20.702	20.713	20.686	20.399	20.445	20.223	21.203	21.105	21.019	20.722	0.335	1.62
7	17.354	17.334	17.377	17.056	17.168	17.274	17.655	17.761	17.656	17.404	0.239	1.37
8	12.617	12.636	12.617	12.225	12.339	12.227	13.033	13.029	13.132	12.651	0.350	2.77

Table S4 The Ct values obtained from serial dilutions (1×10^6 to $1\times10^{0.25}$ $TCID_{50}$/ml) of MRV standard RNA by TaqMan RT-PCR method

$TCID_{50}$/ml (log10)	Ct1	Ct2	Ct3
0.25	36.32	36.44	36.33
0.5	34.95	35.43	35.81
1	33.52	33.36	33.97
2	30.31	30.25	30.29
3	27.11	27.23	27.13

4	24.04	24.12	24.17
5	20.86	20.91	20.84
6	17.68	17.73	17.75

Table S5. Intra-and inter-assay variability of the TaqMan RT-qPCR for MRV detection.

Sample	Intra assay						Inter assay					
	Ct1	Ct2	Ct3	Ct mean	SD	CV%	Ct1	Ct2	Ct3	Ct mean	SD	CV%
1	13.247	13.456	13.566	13.42	0.16	1.2	13.117	13.516	13.668	13.43	0.28	2.06
2	24.536	24.609	24.165	24.44	0.24	1.00	24.763	25.201	24.14	24.70	0.53	2.14
3	28.202	28.203	28.039	28.15	0.09	0.33	29.428	27.754	28.179	28.45	0.87	3.05
4	26.848	26.784	26.991	26.87	0.11	0.29	26.006	27.551	25.832	26.46	0.95	3.60
5	22.911	22.707	21.935	22.50	0.51	2.16	22.732	21.852	23.347	22.64	0.75	3.31
6	19.673	19.509	19.562	19.58	0.08	0.3	19.283	19.579	19.912	19.59	0.31	1.60

SD—Standard deviation. % CV—Percent coefficient variation.

Table S6 The Ct values of different viruses detected by TaqMan RT-PCR method

	MRV-F2B	MRV-SY13	MRV-JS6	MRV-JS1	MRV-XJ23	BADV	BVDV	CEV	CPIV	PPRV	BRV	PRV	COV
Ct1	18.334	14.187	19.631	13.739	22.984	/	/	/	/	/	/	/	/
Ct2	18.379	14.098	19.8376	14.026	23.017	/	/	/	/	/	/	/	/
Ct3	18.865	14.596	19.937	13.925	23.578	/	/	39.974	/	/	/	39.918	/

/: undetected.

Figure S1. Sequence alignment of a conserved fragment of the L1 gene. PCR primers are marked in blue flame, and probe in red.

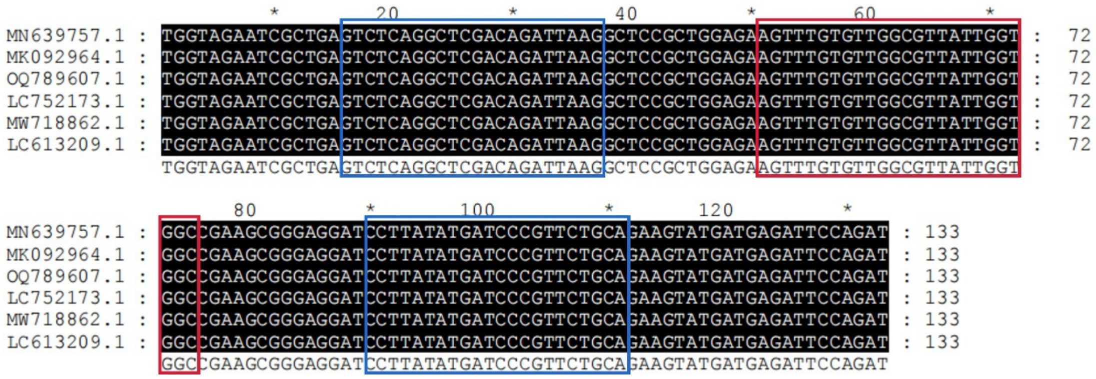

Supplement: Supplementary file 1 [file vetsci-11-00141-s001.zip › vetsci-2866962-supplementary.pdf]
